# The Neutralizing Antibody Response against Severe Acute Respiratory Syndrome Coronavirus 2 and the Cytokine/Chemokine Release in Patients with Different Levels of Coronavirus Diseases 2019 Severity: Cytokine Storm Still Persists Despite Viral Disappearance in Critical Patients

**DOI:** 10.31662/jmaj.2020-0083

**Published:** 2021-01-14

**Authors:** Lidya Handayani Tjan, Tatsuya Nagano, Koichi Furukawa, Mitsuhiro Nishimura, Jun Arii, Sayo Fujinaka, Sachiyo Iwata, Shigeru Sano, Yoshiki Tohma, Yoshihiro Nishimura, Yasuko Mori

**Affiliations:** 1 Division of Clinical Virology, Center for Infectious Diseases, Kobe University Graduate School of Medicine, Kobe, Japan; 2 Division of Respiratory Medicine, Department of Internal Medicine, Kobe University Graduate School of Medicine, Kobe, Japan; 3 Clinical Laboratory Department, Hyogo Prefectural Kakogawa Medical Center, Kakogawa, Japan; 4 Division of Cardiovascular Medicine, Hyogo Prefectural Kakogawa Medical Center, Kakogawa, Japan; 5 Acute Care Medical Center, Hyogo Prefectural Kakogawa Medical Center, Kakogawa, Japan

**Keywords:** COVID-19, critical case, neutralizing antibody, cytokine storm

## Abstract

Patients with coronavirus disease 2019 (COVID-19) exhibit a wide clinical spectrum ranging from mild respiratory symptoms to critical and fatal diseases, and older individuals are known to be more severely affected. The underlying mechanism of this phenomenon is unknown. A neutralizing antibody against viruses is known to be important to eliminate the virus. In addition, this antibody is induced at high levels in patients with severe COVID-19, followed by a termination of virus replication. Severe COVID-19 patients exhibit high levels of cytokines/chemokines, even after the disappearance of the virus. This indicates that cytokines/chemokines play significant roles in disease severity. These findings also suggest that antiviral therapy (monoclonal antibody and/or convalescent plasma therapy) should be administered early to eliminate the virus, followed by steroid treatment after viral genome disappearance, especially in patients with severe symptoms.

In December 2019, a novel coronavirus was identified as the causative agent of a pneumonia outbreak in Wuhan, China ^(1)^. Such virus was named severe acute respiratory syndrome coronavirus-2 (SARS-CoV-2) ^(2)^. Up to now, SARS-CoV-2 has continuously spread worldwide. As of September 30, 2020, there have already been >33 million confirmed cases of SARS-CoV-2 and >1 million deaths worldwide, as reported by the World Health Organization (WHO) COVID-19 dashboard.

The clinical spectrum of the disease caused by SARS-CoV-2 infection, i.e., COVID-19, varies from mild to critical and fatal infections. Older age, hypertension, diabetes mellitus, obesity, malignancy, and several underlying conditions have been found to contribute to the severity of COVID-19 ^(3), (4), (5)^. In addition to these comorbidities associated with COVID-19 prognosis, unique immune responses against SARS-CoV-2 infection among different individuals may result in different clinical manifestations observed in patients.

The results of the investigations of the immune response against SARS-CoV-2 revealed that specific cellular and humoral immunities are induced in COVID-19 patients ^(6)^. Patients with severe infection exhibit a higher antibody titer compared with those with mild infection ^(7)^. The neutralizing antibody titer is also known to be correlated with the severity of COVID-19 patients ^(7), (8)^. Although numerous recent studies used a pseudotyped virus for the neutralizing assay ^(9), (10)^, inconsistent results were observed between neutralizing assays using pseudotyped and authentic viruses ^(11), (12), (13)^. Therefore, for the analysis of the neutralizing activity of serum against SARS-CoV-2, authentic viruses should be utilized.

It has been reported that a hyperinflammmatory state characterized by the cytokine release syndrome (CRS) is associated with the fatalities caused by COVID-19 ^(14)^. Among cytokines and chemokines, interleukin (IL)-6 has been consistently reported to reflect the clinical severity of COVID-19; it has also been considered as the target of therapy in severe cases ^(15), (16)^.

This review summarizes the trend of the neutralizing antibody response against SARS-CoV-2 as well as the cytokine production in COVID-19 patients. In patients with more severe COVID-19, the neutralizing antibody against SARS-CoV-2 is generally induced at a high titer during virus replication. Moreover, the pathogenesis may be affected by the high levels of cytokine/chemokine.

## Neutralizing Antibody Response Against SARS-CoV-2

A neutralizing antibody against a virus is important to eliminate such virus. Several studies have reported that a higher titer of the neutralizing antibody against SARS-CoV-2 is induced in patients with severe infections compared with those with mild infections ^(7), (8), (17), (18)^. The trend of the neutralizing antibody production, cytokine/chemokine levels, and viral disappearance in patients with different levels of COVID-19 severity was also analyzed ^(19)^. In this study, a total of 15 COVID-19 patients were enrolled. All patients were hospitalized at Hyogo Prefectural Kakogawa Medical Center, which is one of the 55 publicly designated medical institutions for infectious diseases in Japan. The patients were diagnosed both clinically and based on the viral genome detection by polymerase chain reaction (PCR). Table 1 presents the demographic and clinical data of the patients.

**Table 1.** Clinical Characteristic of the Patients (Cited and Modified from Tjan LH, Nagano T, Furukawa K, et al. The trend of neutralizing antibody response against SARS-CoV-2 and the cytokine/chemokine release in patients with differing severities of COVID-19: all individuals infected with SARS-CoV-2 obtained neutralizing antibody. medRxiv. 2020 (19). Copyright of the Table Belongs to the Authors).

Code	Sex, Age	Severity^a^	Medical history^b^	Hospitalization duration	Upper respiratory symptom^c^	Pneumonia	Persistent imaging abnormalities	Ventilator introduction (dpo^d^)	Ventilator removal (dpo^d^)	Treatment^e^	Equipment^f^	Outcome
K-Px-10	M, 59	Asymptomatic	DM, glaucoma	20	–	n.d.	n.d.	–	–	–	–	Alive
K-Px-13	M, 54	Asymptomatic	–	8	–	n.d.	n.d.	–	–	–	–	Alive
K-Px-14	F, 54	Asymptomatic	DM	8	–	n.d.	n.d.	–	–	–	–	Alive
K-Px-15	M, 19	Asymptomatic	–	9	–	n.d.	n.d.	–	–	–	–	Alive
K-Px-16	M, 19	Asymptomatic	–	2	–	n.d.	n.d.	–	–	–	–	Alive
K-Px-12	M, 71	Severe	AR, BA, BPH	35	+	+	+	–	–	C	–	Alive
K-Px-1	F, 59	Severe	Cervical cancer, HL, HT, SAS	23	+	+	+	–	–	C, F	–	Alive
K-Px-9	F, 69	Severe	HL, HT	21	+	+	+	–	–	C, F	–	Alive
K-Px-2	M, 57	Critical	–	56	+	+	+	8	33	F, L/R	CHDF, HD	Alive
K-Px-3	M, 74	Critical	DM, glaucoma, HL	44	+	+	+	11	50	C, F, L/R	CHDF	Dead (51 dpo^d^)
K-Px-4	F, 63	Critical	Osteoporosis	80	+	+	+	16	65	C, F, L/R	ECMO	Alive
K-Px-5	M, 65	Critical	HT, hyperuricemia	93	+	+	–	12	32	C, F, L/R	CHDF	Alive
K-Px-6	M, 79	Critical	Arrhythmia, CI, dementia	16	+	+	+	6	15	C, F, L/R	–	Dead (16 dpo^d^)
K-Px-7	M, 78	Critical	AAA, AP, arrhythmia, ASO, CHF, CKD, DM, GB, HT	17	+	+	+	8	21	C, F	–	Dead (22 dpo^d^)
K-Px-8	F, 79	Critical	DM, IgA nephropathy	73	+	+	+	0	36	F, L/R, Steroid (6-9 dpo^d^)	CRRT	Alive

a. The severity criteria were as follows: Mild, upper respiratory symptoms without pneumonia; Moderate, pneumonia requiring no supplemental oxygen; Severe, requirement of supplemental oxygen; Critical, requirement of intensive care, including mechanical ventilation. b. AR, allergic rhinitis; BA, bronchial asthma; BPH, benign prostate hyperplasia; HL, hyperlipidemia; HT, hypertension; SAS, sleep apnea syndrome; DM, diabetes mellitus; CI, cerebral infarction; AAA, abdominal aortic aneurysm; AP, angina pectoris; ASO, arteriosclerosis obliterans; CHF, chronic heart failure; CKD, chronic kidney disease; GB, gallbladder stone. c. Upper respiratory symptom includes fever, cough, and sore throat. d. dpo, days post-onset of symptoms. e. C: ciclesonide, inhaled corticosteroid; F: favipiravir, broad-spectrum antiviral for influenza; L/R: lopinavir/ritonavir, oral antiviral for human immunodeficiency virus; Steroid: steroid pulse with 0.5 g methylprednisolone per day during the indicated period. f. CHDF, continuous hemodiafiltration; HD, hemodialysis; ECMO, extracorporeal membrane oxygenation; CRRT, continuous renal replacement therapy. n.d.: Not determined

Blood samples were collected from each patient. A total of 17 age-matched healthy adults were included in the study as negative controls. They were SARS-CoV-2-specific immunoglobulin G (IgG)-negative medical staff from the same facility we described ^(20)^ and from Kita-Harima Medical Center. An immunochromatographic test kit (2019-nCoV Ab Test; INNOVITA, Hebei, China) was utilized to examine SARS-CoV-2-specific IgG as described ^(20)^. The study was approved by the ethics committee of Kobe University Graduate School of Medicine (approval code: B200200). A written informed consent was also obtained from all the participants.

To evaluate the neutralizing activity of the patients’ serum, we conducted a neutralization test against SARS-CoV-2 (Biken-2 strain) in a biosafety level 3 (BSL3) laboratory. At 24 h before the assay, 4 × 10^4^ VeroE6 (TMPRSS2) cells ^(21)^ per well were seeded into 96-well tissue culture microplates. Duplicate samples of a threefold serial dilution of heat-inactivated (56 °C, 30 min) serum were prepared using Dulbecco’s Modified Eagle Medium. The diluted sera were mixed with 100 50% tissue culture infectious dose (TCID)_50_ of virus and incubated at 37 °C for 1 h. Subsequently, VeroE6 (TMPRSS2) cells in 96-well plates were infected with the serum–virus mixture and incubated at 37°C. After 3 days, these cells were observed under a light microscope, and the neutralizing antibody titer was determined as the highest serial dilution that did not exhibit any cytopathic effects.

**Figure 1.**
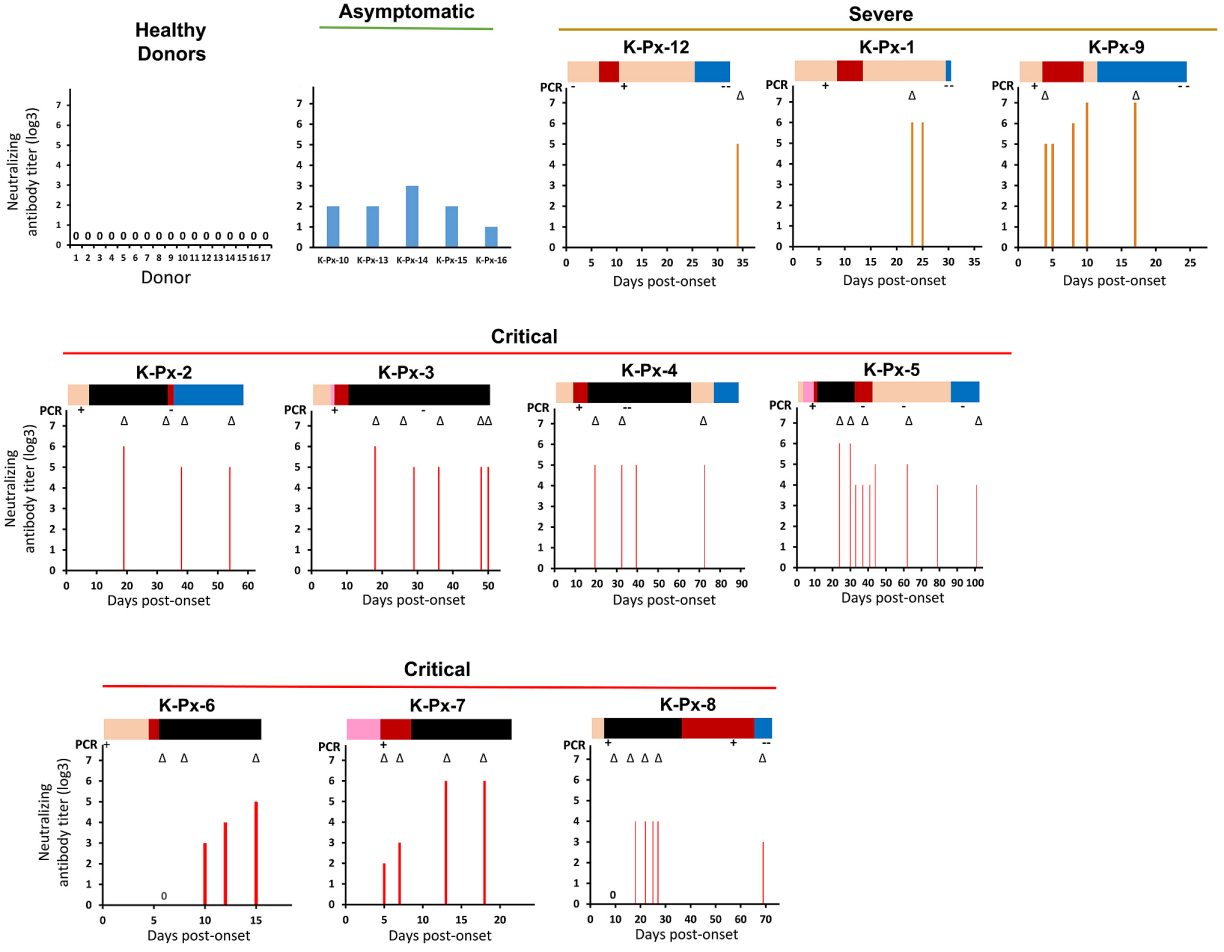
Neutralizing antibody titers of the COVID-19 patients’ sera. (Cited and modified from Tjan LH, Nagano T, Furukawa K, et al. The trend of neutralizing antibody response against SARS-CoV-2 and the cytokine/chemokine release in patients with differing severities of COVID-19: all individuals infected with SARS-CoV-2 obtained neutralizing antibody. medRxiv. 2020 (19). Copyright of the figure belongs to the authors.) The neutralizing antibody titers against SARS-CoV-2 were measured for the patients’ sera. Each panel includes the results for the sera obtained from the indicated patient at a single or several time point(s). The top bars of each panel indicate the symptom transition by colors as follows: *Orange*, upper respiratory symptoms without pneumonia; *Pink*, pneumonia not requiring oxygenation; *Red*, pneumonia requiring oxygenation; *Black*, severe symptoms requiring intensive care including mechanical ventilation; *Blue*, recovered with residual symptoms.**The neutralizing antibody titers of the control sera obtained from 17 healthy donors and 5 asymptomatic patients are also included. The numbers marked as 0 (zero) in the panel indicates that no neutralizing activity has been detected. The open arrowheads below the top bar indicate the time point(s) of cytokine/chemokine measurement presented in Figure 2.

Figure 1 demonstrates that a neutralizing antibody titer against SARS-CoV-2 was detected in all 15 COVID-19 patients, regardless of their disease severity, but not in the healthy controls. These results indicate a highly specific nature of the neutralizing antibody against SARS-CoV-2. The neutralizing antibodies detected in 10 severe/critical COVID-19 patients seemed to have relatively higher titers compared with those detected in the asymptomatic patients ^(19)^. Moreover, Jeewandara et al. reported that severe or moderate COVID-19 patients had higher levels of neutralizing antibodies compared with those with mild COVID-19 or asymptomatic patients ^(18)^. Similarly, Liu et al. observed that COVID-19 patients treated in an intensive care unit had significantly higher peak neutralizing antibody titers compared with those who were not ^(8)^. Based on these reports, including our data, it can be inferred that the neutralizing antibody against SARS-CoV-2 could be induced at higher levels in severe COVID-19 patients.

The results of patients K-Px-6, K-Px-7, K-Px-8, and K-Px-9 revealed that the neutralizing antibody started to be induced around days 4-10 post-onset, and the titer then gradually increased. This suggests that seroconversion occurred around days 4-10 post-onset. Similarly, it has been reported that the neutralizing antibody titer detected in a young man with mild SARS-CoV-2 infection rapidly increased from day 4 until day 20 ^(22)^. In addition, another report indicated that the neutralizing antibody against SARS-CoV-2 was detected at days 10-15 following onset ^(23)^.

To elucidate the effect of the neutralizing antibody production on the persistence of virus replication, the time point(s) at which the neutralizing antibody was initially detected and the virus genome became undetectable was investigated. In seven of the critical patients (excluding two who died in whom a follow-up PCR was not performed), a neutralizing antibody had already been detected at the time point at which the virus genome was not detected by PCR. In patient K-Px-8, a different pattern was observed; the viral genome could still be detected in this patient at day 56 post-onset, even though the neutralizing antibody had already been induced. Of note, patient K-Px-8 was the only critical patient in this study who received corticosteroid pulse therapy from the disease onset.

## The Trend of Cytokine/chemokine Expression in Patients with Different Levels of COVID-19 Severity

**Figure 2.**
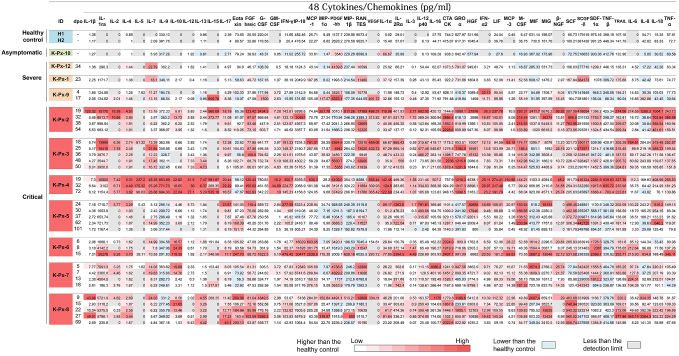
Cytokine/chemokine concentrations in the sera obtained from COVID-19 patients (Cited and modified from Ref 19. Copyright of the figure belongs to the authors.) The serum levels of cytokines and chemokines were evaluated using the multiplex suspension array system Bio-Plex Pro™ Human Cytokine Screening 48-Plex Panel (Bio-Rad). Each of the observed fluorescent values for 48 kinds of cytokines/chemokines was blank-corrected and translated to the concentration using the respective standard curves for each cytokine or chemokine. Subsequently, the values were used to calculate the serum concentration by considering the dilution rate. The values are the averages of the duplicated wells. Values lower than the applicable range of the standard curve were shown as zero and indicated in *gray*. Values lower than the averages of those of the healthy controls are indicated in pale blue. The values higher than the average are indicated in red color according to the levels.

Cytokine release has been demonstrated to contribute to the severity of COVID-19 ^(14), (15)^. Therefore, the levels of cytokines, chemokines, and growth factors in the sera obtained from patients with different levels of COVID-19 severity were investigated at various time points as described ^(19)^. A total of 48 cytokines, chemokines, and growth factors were measured using the Bio-Plex Pro™ Human Cytokine Screening 48-Plex Panel (Bio-Rad, Hercules, CA) following the manufacturer’s instructions. Figure 2 presents the measured levels of cytokines, chemokines, and growth factors in the sera obtained from the patients, all of which are expressed in picograms per milliliter of serum. To visualize the trend of the individual cytokine expression in the different disease severity groups, a graded color scale (heat map) of the values of cytokine, chemokine, and growth factor was created. In two healthy controls, the average measured values for cytokine, chemokine, and growth factor were also determined. The values higher than the control values are indicated in red according to the amount.

A total of six out of the seven critical patients required mechanical ventilation support after several days of hospitalization. During treatment, disease progressivity may continue, and the day of ventilator introduction may therefore reflect the time point at which the disease clinically progresses to a more severe condition. In addition, the day of ventilator removal may reflect the time point at which the patient exhibits sufficient clinical improvement in respiratory system function. We focused on the kinetics of cytokine/chemokine release in relation to the patients’ clinical presentations. In the sera obtained from patient K-Px-2, the levels of several cytokines and chemokines were higher during the period of his ventilator usage (days 8-33 post-onset)―especially in the earlier days―compared with the levels beyond this period. In three other critical COVID-19 patients (K-Px-4, K-Px-5, and K-Px-8), all of whom recovered, a similar trend was observed. The single exception was patient K-Px-4, whose cytokine and chemokine levels were constantly high for an extended period of time, including after her recovery period.

The trend of the cytokine/chemokine release of three critical COVID-19 patients who died (K-Px-3, K-Px-6, and K-Px-7) was unique in each patient. Patient K-Px-3 exhibited consistently high levels of most of the cytokines and chemokines from 1 week after the start of ventilator use until his death (day 18 to day 50 post-onset) with the following exceptions, which exhibited a reducing trend until death: IL-1ra, IL-5, IL-17, monocyte chemoattractant protein (MCP)-1, vascular endothelial growth factor (VEGF), IL-12p40, IL-16, hepatocyte growth factor (HGF), interferon (IFN)-α2, MCP-3, IL-6, IL-18, and tumor necrosis factor (TNF)-α. Contrarily, the sera obtained from patient K-Px-6 exhibited an increased level of most of the cytokines and chemokines in the days before his death. These results indicate that the “cytokine storm” was the possible cause of death. Patient K-Px-7, who had more comorbidities related to COVID-19 severity compared with the other patients, exhibited a third pattern; he did not demonstrate an increasing trend of cytokines or chemokines until his death.

Of note, clearly higher amounts of most cytokines and chemokines were observed in critical COVID-19 patients compared with patients with milder symptoms at all time points. This observation is in agreement with the reports that the cytokine storm contributes to the severity of COVID-19 ^(24)^. IL-6 plays a significant role in the cytokine storm ^(15)^ and signals through two main pathways (cis- and trans-signaling pathways), which also play a major role in the CRS ^(14), (25)^. Consistent with earlier reports, the results of our present analyses revealed that the amount of IL-6 in the sera clearly increased in all 10 of the severe/critical COVID-19 patients, but not in the asymptomatic patients.

Although in the present study, the measured levels of most of the cytokines and chemokines were higher in critical COVID-19 patients compared with those with milder symptoms, high levels of some of the cytokines and chemokines, namely, IL-7, platelet-derived growth factor (PDGF)-BB, regulated on activation, normal T cell expressed and secreted (RANTES), IFN-α2, and stem cell growth factor (SCGF)-β, were detected in some of the patients with milder symptoms. This result is inconsistent with an earlier report, which indicated that PDGF-BB, RANTES, and IL-9 were consistently low in patients with severe and fatal infections compared with the mild-infection group ^(26)^. In our present series, the levels of RANTES and PDGF-BB were not consistently higher in the non-severe COVID-19 patients compared with the severe COVID-19 patients. Contrarily, IL-9 has been consistently detected at values comparable to those of the healthy controls in all of the patients, regardless of the disease severity, except for patient K-Px-4. This patient exhibited relatively high levels of most of the cytokines and chemokines over an extended period of time, including the recovery phase.

## In Severe COVID-19 patients, the Virus Disappears after the Induction of the Neutralizing Antibody, but the Cytokine Storm Continues

Several studies and our present investigation have demonstrated that a high titer of the neutralizing antibody is induced in COVID-19 patients with severe symptoms compared with those with mild symptoms. The possible effect of the induction of the neutralizing antibody to eliminate the virus was analyzed. Our findings revealed that the neutralizing antibody was consistently induced before the SARS-CoV-2 genome was undetectable, thus suggesting an efficient viral clearance by a successful induction of neutralizing antibodies. Of note, the level of numerous cytokines, chemokines, and growth factors were still high at the time point at which a viral genome PCR had presented a negative result. This result suggests that the cytokine storm continues even after the disappearance of the virus, which affects the severity of COVID-19.

Based on the results of our analyses of the courses of the COVID-19 patients’ clinical conditions and their neutralizing antibody titers and cytokine/chemokine levels, we suggest the following concept. In severe COVID-19 patients, SARS-CoV-2 abundantly or systemically replicates, especially in the lungs. The higher positivity rate of detecting SARS-CoV-2 in the sputum compared with nasopharyngeal or throat swabs may be explained by the efficient replication of this virus in the lungs ^(27), (28), (29), (30)^. Also, innate immunity is induced in response to viral replication, followed by the induction of acquired immunity, which can be demonstrated by the detection of a specific antibody and T lymphocytes recognizing SARS-CoV-2. The induction of innate immunity is reflected in the increased levels of cytokines and chemokines, which are responsible for the occurrence of cytokine storm. Finally, both virus replication and cytokine storm causes lung damage in patients with severe disease.

The induction of acquired immunity may then efficiently eliminate the virus, as the viral genome becomes undetected at the time point at which the neutralizing antibody has reached a higher level. However, by the time acquired immunity is induced, the disease may still be progressing, and lung damage may persist as the cytokine storm remains uncontrolled.

## Conclusion

High levels of neutralizing antibodies against SARS-CoV-2 were detected in the sera obtained from severe/critical COVID-19 patients. This indicates that abundant virus replication occurred in their bodies. Substantial productions of several types of cytokines/chemokines were induced in critical COVID-19 patients. This indicates that the high expression of cytokines/chemokines contributes to the disease severity. Therefore, both the virus replication at the earlier stage and the cytokine storm that persists after the virus disappearance may cause lung damage in patients with critical COVID-19. Based on this understanding, we suggest that therapy using convalescent plasma or a monoclonal antibody and/or an effective antiviral agent (when available) may provide better outcomes when administered earlier, before the initiation of severe cytokine production. A similar recommendation has also been provided elsewhere ^(31)^. When viral clearance is confirmed, steroid therapy could be administered in severe cases to suppress the cytokine storm if observed.

## Article Information

### Conflicts of Interest

None

### Sources of Funding

This work was supported by Hyogo Prefectural Government.

### Acknowledgement

We thank Kazuro Sugimura MD, PhD (Executive Vice President, Kobe University) for his full support to promote this study. We also thank Yukiya Kurahashi, Zhenxiao Ren, Anna Lystia Poetranto, Salma Aktar, Jing Rin Huang, and Silvia Sutandhio (Division of Clinical Virology, Center for Infectious Diseases, Kobe University Graduate School of Medicine) for technical support in this study. We express our sincere gratitude for cooperation and participation of staffs of Hyogo Prefectural Kakogawa Medical Center. We thank Research Foundation for Microbial Diseases of Osaka University (BIKEN), Osaka University for providing SARS-CoV-2 strain.

### Authors of Contributions:

Lidya Handayani Tjan and Tatsuya Nagano contributed equally to the work.

### Disclaimer

Yasuko Mori is one of the Editors of JMA Journal and on the journal's Editorial Staff. She was not involved in the editorial evaluation or decision to accept this article for publication at all.
